# Predictors of shorter sleep in early childhood

**DOI:** 10.1016/j.sleep.2014.01.005

**Published:** 2014-05

**Authors:** Laura McDonald, Jane Wardle, Clare H. Llewellyn, Cornelia H.M. van Jaarsveld, Abigail Fisher

**Affiliations:** Health Behaviour Research Centre, Department of Epidemiology and Public Health, University College London, Gower Street, London WC1E 6BT, UK

**Keywords:** CI, confidence interval, OR, odds ratio, SD, standard deviation, m, minutes, Sleep, Child, Predictors, Behaviour, Bedtime, Wake time

## Abstract

**Objective:**

The aim of this study was to identify socio-demographic and home environmental predictors of shorter sleep in early childhood, and to examine whether effects were mediated by the timing of bedtime or wake time.

**Methods:**

Participants were from Gemini, a British birth cohort of twins, and included 1702 children; one randomly selected from each twin pair. Parents reported night-time sleep duration at an average age of 15.8 months (range 14–27 months) using a modified version of the Brief Infant Sleep Questionnaire. Multiple logistic regression models were used to identify predictors of shorter sleep for this study.

**Results:**

Using a cut-off of <11 h a night, shorter sleep was reported in 14.1% of children. Lower maternal education, non-white ethnic background, being male, low birth weight, living in a home with >1 older child and watching >1 h of TV in the evening were independently associated with shorter sleep. Mediation analyses showed that associations between education, ethnicity, evening TV viewing and sleep were driven predominantly by later bedtimes, while sex differences were driven predominantly by earlier wake times in boys.

**Conclusion:**

In this sample, multiple environmental factors were associated with shorter sleep in young children, with several operating predominantly through later bedtime. An emphasis on the importance of an early and consistent bedtime could help promote healthy sleep and reduce inequalities in child health.

## 1 Introduction

In early childhood, insufficient sleep at night can impair learning and memory [1] and disrupt emotional regulation [2]. It is increasingly being shown to be associated with adverse health outcomes, notably an increased risk of obesity [3]. The health implications of short sleep make it important to understand its determinants, particularly in early life when patterns of sleep behaviour may be being established [4].

There are currently no clinical recommendations to define optimal sleep in early childhood [5], but sleeping <11 h per night substantially increases the risk of obesity in children under 5 years of age [3,6]. Between 18 and 24 months of age, children sleep on average 11.3 h a night and 1.5 h during the daytime [7]. However, daytime and night-time sleep may serve different functions; daytime sleep is not associated with obesity risk in children for example [6,8]; and they have different predictors [9].

Twin studies suggest that night-time sleep duration in early childhood is largely determined by the shared environment [10,11]. Multiple factors within the home have been shown to influence sleep. In an sample of over 5000 children aged from birth to 36 months, a regular bedtime routine and limited TV exposure were among the strongest predictors of longer sleep at night [9]. There are also marked ethnic and socio-economic differences in the sleep duration of young children [12], probably because these factors affect attitudes and practices surrounding sleep behaviours [13]. In a sample of over 10,000 children aged 18 months from the Avon Longitudinal Study of Parents and Children (ALSPAC), those from non-white ethnic backgrounds slept significantly less than other children in the cohort [7]. Other studies have also shown that children from non-white families, or those where the mother has less education, report shorter sleep at night [14,15], with some evidence that differences persist through the lifecourse [16]. Although multiple features within a child’s physical and social environment have been associated with night-time sleep duration, it is not known whether these effects are mediated by the timing of bedtime or wake time. This information could help to understand the mechanisms of shorter sleep and inform strategies to improve sleep in early life.

This study therefore aimed to test whether predictors of shorter sleep at night operated primarily through sleep initiation (bedtime) or sleep termination (wake time).

## 2 Participants and methods

The Gemini study, described in full elsewhere [17], is a British twin birth cohort established to investigate genetic and environmental determinants of health in early life. The baseline sample includes 2402 families with twins born in the UK between March and December 2007 (36% of all live twin births). The proportion of male, female and opposite sex twin pairs in this sample is comparable to UK twin statistics. The geographic distribution of Gemini families reflects the UK population density, although white-British families from higher socio-economic backgrounds are over-represented [17]. The second wave of data collection, which included assessments of sleep, was completed by 1931 families (80% of baseline sample). For the present analyses, one child from each family was randomly selected to avoid non-independence of data. Written informed consent was provided by all parents. Ethical approval was granted by the University College London Committee of non-National Health Service Human Research.

### 2.1 Sleep behaviour

Sleep was assessed with a modified version of the Brief Infant Sleep Questionnaire [18] when the children were on average 15.8 months old. The primary caregiver reported on several aspects of sleep behaviour including daytime sleep, whether their child regularly woke up at night (yes/no), bedtime and wake time. Night-time sleep duration was calculated from parent-reported bedtime and wake time; a method that has been validated against objective measures [18]. In a subsample of 40 families, 1 week test–retest reliability of the sleep questionnaire was high (e.g., intraclass correlation 0.89; 95% confidence interval (CI) 0.76–0.95 for night-time sleep duration).

There are currently no clinical recommendations to define short sleep in early childhood, but population data can provide an indication of basic sleep requirements [19]. At around 18 months of age, age-specific reference values suggest children sleep on average 11.3 (standard deviation (SD) 1.3) h per night [7], although individual variability exists. Sleeping <11 h per night at this age is also associated with an increased risk of obesity [3,6], therefore reflecting a level of sleep that is not sufficient to support optimal functioning or health. Therefore, shorter sleep in this study was defined as sleep <11 h per night [3]. We also conducted a sensitivity analysis using a cut point at the 10th percentile of sleep duration (<10.5 h per night) and patterns of results were the same, so data are presented using <11 h as the demarcation for short sleep.

### 2.2 Socio-demographic and environmental characteristics

Socio-demographic risk factors were reported by the primary caregiver in baseline questionnaires. These included maternal education (up to secondary school, college and beyond), maternal ethnicity (white and non-white) and child sex (male and female). Birth weight was recorded by asking parents to photocopy or transcribe health records and was categorised as low (⩽2500 g) or normal weight (>2500 g) [20]. Environmental factors reported at the second wave of data collection when the children were 15.8 months old included the number of older children living in the home (0, 1 and >1), and hours of TV viewing in the morning and after 6:30 in the evening (⩽1 and >1 h).

### 2.3 Statistical analyses

Logistic regression models were used to identify significant predictors of shorter sleep. Models were first run separately for each factor, followed by a multiple logistic regression model. All models were adjusted for age because the range in the sample (14–27 months) was too narrow to study age effects, and for daytime sleep; because at this age daytime sleep still represents a large proportion of total sleep duration that a child receives [19], and shows considerable ethnic variation [21]. All models were also adjusted for regular night waking, as this may disrupt the normal sleep–wake cycle.

To test whether the predictors of shorter night-time sleep operated primarily through wake time or bedtime, a standard, four-step mediation approach was used [22]. Mediation is considered to take place when (1) the mediator (wake time or bedtime) significantly predicts the dependent variable (shorter sleep), (2) the independent variable significantly predicts the dependent variable, (3) the independent variable significantly predicts the mediator and (4) the association between the independent variable and the dependent variable is substantially reduced when the mediator is included in the model. Criteria 1 and 2 were assessed with the logistic regression and multiple logistic regression models used to identify predictors of shorter sleep. Criterion 3 was assessed using two separate multiple linear regression models predicting wake time and bedtime. Criterion 4 was assessed using two separate multiple logistic regression models including all determinants and either wake time or bedtime. Where the results satisfied criteria for mediation, the Sobel test was used as the test of significance [23].

## 3 Results

Complete data were available for 1702 children (71.0% of baseline sample). The average night-time sleep duration in the sample was 11.6 h per night (SD 52 min) and the average daytime sleep was 1.9 h (SD 41 min). Shorter sleep (<11 h per night) was reported in 14.1% of the sample. There was no significant association between daytime and night-time sleep duration, indicating that shorter night-time sleepers are not compensating during the day. Table 1 shows the percentage of children experiencing shorter night-time sleep by family characteristics. Shorter night-time sleep was more common in children from ethnic minority families and families where the mother had less education. Shorter sleep was also more common in boys, and children who watch more than 1 h of TV in the morning or evening.

The average bedtime of the shorter-sleeping group was 8:05 pm (SD 61 min) compared with 7:04 pm (SD 33 min) in those with ⩾11 h of sleep. The average wake time of the shorter-sleeping group was 6:14 am (SD 54 min) compared with 6:56 am (SD 39 min) in those with ⩾11 h of sleep. There was a positive correlation between bedtime and wake time (*r *= 0.29, *p *< 0.001).

### 3.1 Predictors of shorter sleep

Table 1 presents the logistic regression models predicting shorter sleep. Lower maternal education (odds ratio (OR) = 1.64, 95% CI 1.23–2.17), coming from a minority ethnic background (OR = 5.10, 95% CI 3.16–8.24), being male (OR = 1.45, 95% CI 1.08–1.92) and having been born at a low birth weight (OR = 1.43, 95% CI 1.07–1.90) significantly increased the odds of shorter sleep. Having more than one older child in the home (OR = 1.70, 95% CI 1.17–2.47) and watching more than an hour of TV in the morning (OR = 1.47, 95% CI 1.10–1.96) or evening (OR = 2.22, 95% CI 1.55–3.18) were also associated with shorter sleep. In the multiple logistic regression with all variables included in the analysis, the effect of morning TV was no longer significant, but all other effects remained significant (Model 2 in Table 1).

### 3.2 Pathways to shorter sleep

As expected, because night-time sleep is a function of both bedtime and wake time, children who woke up later in the morning (OR = 0.22, 95% CI 0.17–0.29) were less likely to experience shorter sleep, and those who went to bed later in the evening were substantially more likely to be shorter sleepers (OR = 6.04, 95% CI 4.72–7.72). Table 2 shows associations with wake time and bedtime for all variables that independently predicted shorter sleep. The only predictor of earlier waking was being male; other effects were either non-significant or associated with ‘later’ wake times. By contrast, lower maternal education, ethnic minority status and watching >1 h of evening TV were associated with a later bedtime.

The results of adding either wake time (Model 1) or bedtime (Model 2) into the multiple logistic regression model predicting shorter sleep are shown in Table 3. These can be compared with the ORs shown in Table 1 (Model 2). After including wake time, all determinants remained significant predictors of shorter sleep with the exception of sex. The Sobel test showed significant mediation of the gender effect by wake time (*p *< 0.001). Other determinants did not satisfy the criteria for mediation by wake time.

Including bedtime in the model reduced the ORs associated with maternal education, ethnicity and time spent watching TV in the evening to non-significance. Sobel tests indicated a later bedtime significantly mediated the associations between lower education (*p *< 0 .001), ethnic minority status (*p *< 0.001) and evening TV viewing (*p *< 0.001) and shorter sleep. Other determinants did not satisfy the criteria for mediation by bedtime.

## 4 Discussion

This study helps to establish predictors of shorter night-time sleep in early life and identifies several key influences that operate predominantly through later bedtime. Understanding the importance of an early and consistent bedtime could help to promote healthy sleep and reduce inequalities in child health.

Ethnicity and maternal education emerged as significant influences on shorter sleep in early childhood. Children living in non-white families or families with lower maternal education were more likely to sleep for <11 h per night. This finding supports a body of literature citing sociocultural differences in sleep behaviour [24,12], and emerges probably because both maternal education and ethnicity affect multiple features of the child’s physical and social environment, including parental attitudes and practices surrounding sleep behaviour [13,14,25]. Importantly, we found that ethnic differences in sleep were partly explained by later bedtimes in ethnic minority groups. Bedtime practices are often culturally defined [12], and our results indicate that they may drive disparities in sleep behaviour. Interestingly, children from ethnic minority backgrounds also reported later morning wake times, suggesting a tendency towards a later sleep schedule or propensity to offset sleep loss at night. Maternal education differences were also driven principally by later bedtimes in the lower education group with no differences in wake times. While education and ethnicity are established risk factors for shorter sleep at night [12,26], they are not themselves modifiable; these groups might need specific emphasis on the importance of an early bedtime in determining sleep in early life.

Children who watched more than an hour of TV in the evening were also substantially more likely to be shorter sleepers. This supports previous research demonstrating that longer periods of TV exposure are predictive of greater bedtime resistance, more difficulty maintaining sleep and shorter sleep duration [14,26,27]. Morning TV viewing did not independently predict shorter sleep. While evening TV viewing may be partly a compensating activity for sleep, it could also impair a child’s ability to initiate sleep by raising levels of arousal before bedtime. Importantly, later bedtimes also accounted for the association between time spent watching TV in the evening and risk of shorter sleep. Children who watch more TV in the evening also woke up significantly later in the morning, possibly indicating a tendency to compensate for sleep lost at night, although it could reflect broader family patterns. However, the tendency towards later morning wake times was insufficient to offset sleep lost at night. Children under 2 years are recommended not to watch any TV [28]; however, many parents may incorporate TV viewing into their child’s bedtime routine [25]. Engaging in bedtime practices that do not include TV exposure could help children maintain an early bedtime and encourage healthy sleep during early childhood.

The importance of an early bedtime is emphasised in many ‘sleep hygiene’ recommendations. They often advocate that children maintain a bedtime before 9 pm, a consistent bedtime routine, and sleep independently in a quiet, dark room without the presence of media devices [29]. In the present study, even children in the shorter sleeping group went to bed well before 9 pm, suggesting these recommendations may need to be amended for young children who require more sleep at night and may not consistently compensate by waking later or sleeping longer during the day.

Although several domains of the environment influence sleep in early life, not all operate in the same way. We found that boys were more likely to experience shorter sleep than girls and this seemed to be due largely to earlier wake times in boys. Gender-specific differences in sleep patterns have been reported previously [7], with some evidence suggesting males are more likely to sleep for shorter periods at night and experience a lower percentage of motionless or quiet sleep [15,30]. Birth weight and the number of older children living in the home also emerged as independent predictors of shorter sleep, but appeared to exert a more general influence on sleep duration and were not associated with either bedtime or wake time. Living in a home with more people is associated with a greater level of chaos [31], so this could disrupt either end of the night-time sleep episode.

Importantly, all predictors emerged independently of daytime sleep. Although daytime sleep still represents a large proportion of total sleep time in this age group [7], daytime sleep did not differ significantly between shorter and longer sleepers. Young children may not always compensate sufficiently for shorter sleep at night by sleeping for longer periods during the day, further underlying the importance of an early bedtime and obtaining sufficient sleep at night.

### 4.1 Limitations

Several limitations should be considered when interpreting the results. First, parent-reported sleep in early childhood is a limitation. However, this method is common in larger, population-based studies and may even provide a better representation of habitual sleep behaviour than a brief period of objective recording [18]. Encouragingly, the mean sleep duration in this sample (11.6 h per night) is comparable to studies of singletons citing references values for normative sleep behaviour [19]. The use of twins may be a limitation, and should be considered when interpreting effects related to the number of siblings present in the home. However, it is reassuring that sleep duration is comparable across singletons and twins as this suggests twins are not keeping one another awake at night.

Although bedtime itself is a component from which sleep duration is calculated, our sleep duration variable was dichotomised and neither bedtime nor wake time alone was used to define shorter sleep. Factors associated with shorter sleep also did not show the same pattern of association with bedtime and wake time, indicating that these variables are to some extent under separate influence. As there are no clinical recommendations to define short sleep, it is important to note that patterns of results were the same when the demarcation of short sleep was set below the 10th percentile of sleep duration. While our sample was approximately representative of the UK population, the ethnic minority proportion was relatively small. Therefore, we were unable to examine differences between different ethnic minority groups; this would require a sampling method that focussed specifically on ethnic grouping.

## 5 Conclusion

Multiple factors within the home environment are associated with shorter sleep in early childhood. Some operate predominantly through later bedtime (ethnicity, maternal education and evening TV), others on earlier wake time (gender) and others have a more general influence on sleep duration (birth weight and older children in the home). In this sample, lower maternal education, ethnic minority status and evening TV viewing, which were among the strongest influences, were associated with shorter sleep through later bedtime. This suggests that bedtime could represent a modifiable target for interventions designed to improve sleep during early life.

## Conflict of interest

The ICMJE Uniform Disclosure Form for Potential Conflicts of Interest associated with this article can be viewed by clicking on the following link: http://dx.doi.org/10.1016/j.sleep.2014.01.005.

Conflict of interest ICMJE Form for Disclosure of Potential Conflicts of Interest form.

## Figures and Tables

**Table 1:** Percentage of participants reporting shorter sleep by family characteristics and logistic regression models predicting shorter night-time sleep.

Risk factors	Shorter sleep	Model 1	Model 2
	(<11 h)	(Simple logistic regression)	(Multiple logistic regression)
	*n* (%)	OR (95% CI)	OR (95% CI)
Total (*n = *1702)	240 (14.1)		

Maternal education			
High (*n = *964)	108 (11.2)	1.00	1.00
Low (*n = *735)	132 (17.9)	1.64 (1.23–2.17)⁎⁎	1.46 (1.07–1.99)⁎

Ethnicity			
White (*n = *1615)	200 (12.4)	1.00	1.00
Non-white (*n = *87)	40 (46.0)	5.10 (3.16–8.24)⁎⁎	5.05 (3.08–8.27)⁎⁎

Sex			
Female (*n = *874)	104 (11.9)	1.00	1.00
Male (*n = *828)	136 (16.4)	1.45 (1.08–1.92)⁎	1.61 (1.19–2.17)⁎

Birth weight (g)			
>2500 (*n = *858)	105 (12.2)	1.00	1.00
⩽2500 (*n = *844)	135 (16.0)	1.43 (1.07–1.90)⁎	1.45 (1.07–1.96)⁎

Older children			
0 (*n = *889)	121 (13.6)	1.00	1.00
1 (*n = *563)	66 (11.7)	0.84 (0.61–1.17)	0.83 (0.58–1.17)
>1 (*n = *250)	53 (21.2)	1.70 (1.17–2.47)⁎	1.58 (1.06–2.35)⁎

Morning TV (h)			
⩽1 (*n = *1176)	141 (12.0)	1.00	1.00
>1 (*n = *526)	99 (18.8)	1.47 (1.10–1.96)⁎	1.13 (0.80–1.58)

Evening TV (h)			
⩽1 (*n = *1489)	183 (12.3)	1.00	1.00
>1 (*n = *213)	57 (26.8)	2.22 (1.55–3.18)⁎⁎	1.89 (1.26–2.84)⁎

Mediators			
Wake time (per hour)	–	0.22 (0.17–0.29)⁎⁎	–
Bedtime (per hour)	–	6.04 (4.72–7.72)⁎⁎	–

Abbreviations: OR, odds ratio; CI, confidence interval. Model 2 is a multiple logistic regression model containing all risk factors predicting shorter sleep. All models adjusted for age, daytime sleep and regular night waking.

⁎ *p* < 0.05.

⁎⁎ *p* ⩽ 0.001.

**Table 2:** Multiple linear regression model predicting wake time and bedtime.

	Wake time		Bedtime	
Risk factors	B (SE)	*p*-value	B (SE)	*p*-value
Maternal education				
Low vs. high	0.04 (0.04)	0.226	0.11 (0.03)	0.001

Ethnicity				
Non-white vs. white	0.31 (0.08)	<0.001	0.80 (0.08)	<0.001

Sex				
Male vs. female	−0.16 (0.03)	<0.001	0.02 (0.03)	0.529

Birth weight (g)				
⩽2500 vs. > 2500	0.02 (0.04)	0.643	0.01 (0.03)	0.768

Older children				
>1 vs. 0 vs. 1	−0.02 (0.02)	0.355	−0.03 (.02)	0.259

Evening TV (h)				
>1 vs.⩽1	0.17 (0.05)	0.001	0.41 (0.05)	<0.001

Abbreviations: B = unstandardised regression coefficient; SE = standard error. Models adjusted for age, daytime sleep and regular night waking.

**Table 3:** Logistic regression models predicting shorter sleep including either wake time (Model 1) or bedtime (Model 2).

Risk factors	Model 1 (including wake time)	Model 2 (including bedtime)
	OR (95% CI)	OR (95% CI)
Maternal education		
High	1.00	1.00
Low	1.55 (1.10–2.17)⁎	1.16 (0.82–1.64)

Ethnicity		
White	1.00	1.00
Non-white	12.63 (6.95–22.94)⁎⁎	1.73 (0.92–3.26)

Sex		
Female	1.00	1.00
Male	1.24 (0.89–1.72)	1.73 (1.23–2.43)⁎

Birth weight (g)		
>2500	1.00	1.00
⩽2500	1.46 (1.05–2.04)⁎	1.52 (1.08–2.14)⁎

Older children		
0	1.00	1.00
1	0.79 (0.54–1.16)	1.01 (0.67–1.48)
>1	1.79 (1.15–2.79)⁎	1.34 (0.83–2.16)

Evening TV (h)		
⩽1	1.00	1.00
>1	3.02 (1.97–4.64)⁎⁎	1.06 (0.67–1.66)

Abbreviations: OR, odds ratio; CI, confidence interval. Model 1 is a multiple logistic regression model including all risk factors and wake time predicting shorter sleep. Model 2 is a multiple logistic regression model including all risk factors and bedtime predicting shorter sleep. All models are adjusted for age, daytime sleep and regular night waking.

⁎ *p* < 0.05.

⁎⁎ *p* ⩽ 0 .001.
